# Age-based prediction of incidence of complications during inpatient stroke rehabilitation: a retrospective longitudinal cohort study

**DOI:** 10.1186/1471-2318-14-41

**Published:** 2014-03-31

**Authors:** Chien-Min Chen, Hung-Chih Hsu, Chia-Hao Chang, Chu-Hsu Lin, Kai-Hua Chen, Wei-Chi Hsieh, Wen-Ming Chang

**Affiliations:** 1 Department of Physical Medicine and Rehabilitation, Chang Gung Memorial Hospital, Chiayi. No.6, W. Sec., Jiapu Rd., Puzih City, Chiayi County 613, Taiwan; 2 School of Medicine, Chang Gung University, 259 Wen-Hwa 1st Road, Kwei-Shan, Tao-Yuan County 333, Taiwan; 3 Graduate Institute of Clinical Medical Sciences, College of Medicine, Chang Gung University, No.259, Wen-Hwa 1st Road, Kwei-Shan, Tao-Yuan County 333, Taiwan; 4 Department of Nursing, Chang Gung University of Science and Technology, Chiayi Campus. No.2, W. Sec., Jiapu Rd., Puzih City, Chiayi County 613, Taiwan; 5 Department of Physical Medicine and Rehabilitation, Chang Gung Memorial Hospital, Yunlin. No.707, Gongye Rd., Mailiao Township, Yunlin County 638, Taiwan

**Keywords:** Age, Stroke, Complications, Rehabilitation

## Abstract

**Background:**

Stroke complications can occur not only in the acute ward but also during the subsequent rehabilitation period. However, existing studies have not adequately addressed the incidence of various complications among stroke in patients undergoing rehabilitation using a longitudinal method. We aimed to investigate the longitudinal impact of age on complication rates in patients undergoing inpatient stroke rehabilitation at different disease stages.

**Methods:**

Five hundred and sixty-eight first-time stroke patients transferred to the rehabilitation ward between July 2002 and June 2012 were included in the study. Patients were stratified into age groups for comparison: <65 years (young), 65 years to <75 years (younger old), and ≥75 years (older old). In total, 30 different complication types were recorded for analysis.

**Results:**

Constipation, shoulder pain, symptomatic urinary tract infection (UTI), and fever were common complications during initial stay in the rehabilitation ward, and incidence was >10% in all three age groups. The frequency of incidence of upper gastrointestinal bleeding (UGIB) was higher in the younger old (17.9%) and older old (20.6%) groups than in the young group (4.1%) during initial stay in the rehabilitation ward (p < 0.001). The incidence of UGIB was higher in the younger old (8.04%) and older old (8.33%) groups than in the young group (0.19%) during subsequent stay in the rehabilitation ward (p = 0.011). The incidence of symptomatic UTI was higher in the younger old (21.0%) and older old (20.0%) groups than in the young group (11.5%) during initial stay in the rehabilitation ward (p = 0.019). The incidence of symptomatic UTI was higher in the older old group (29.17%) than in the younger old (9.21%) and young (3.14%) groups during subsequent stay in the rehabilitation ward (p < 0.001).

**Conclusions:**

Age does not affect every complication type. UGIB and symptomatic UTI occurred more frequently in stroke patients aged ≥65 years during their stay in the rehabilitation ward.

## Background

Stroke is a leading cause of long-term disabilities with respect to balance, movement, speech, swallowing, urination, and defecation, which has become a global public health concern. Complications occur not only in the acute ward [1,2] but also during the subsequent rehabilitation period [3,4]. Functional improvement may occur following rehabilitation, but stroke-related complications are considered to potentially influence functional outcome [5].

It has been reported that 44%–75% [6,7] of stroke patients experience at least one complication during inpatient rehabilitation, the most common being musculoskeletal pain [3,8,9], depression [3,4,7,8,10], fever [11], pressure ulcer [4,8,9], infection [7,12], fall [4,7,8,13], upper gastrointestinal bleeding (UGIB) [6]– [8], seizure [4,7,8,12], nutritional deficiency [7], deep venous thrombosis [3,7,9,13], stroke progression [6,9,13], and pulmonary embolism [7].

Although older age is associated with increased stroke incidence [14,15], its effect on the incidence of complications during rehabilitation remains controversial. Doshi et al. demonstrated no significant difference in the frequency of common complications between groups aged ≥65 and <65 years among stroke patients transferred to rehabilitation wards [10]. In Kuptniratsaikul’s study [3] that enrolled 327 patients with stroke from nine inpatient rehabilitation centers, age >60 years was not associated with complications. However, in a subsequent study [16] that followed 214 stroke patients during the first year after discharge from rehabilitation wards, age >60 years was found to be the only key factor associated with complications. Among elderly (age ≥65 years) inpatients with acute first-time stroke who underwent rehabilitation, older age was an important risk factor for predicting UGIB [17]. Kwan et al. [18] reported that age may increase the risk of overall infection. However, another study [19] focusing on stroke patients aged ≥65 years revealed that older age was not a risk factor for the occurrence of infection among patients receiving inpatient rehabilitation.

Although some stroke patients may be hospitalized in the rehabilitation ward more than once for post-stroke rehabilitation, existing studies using a longitudinal method have not adequately addressed the incidence of various complications among stroke inpatients undergoing rehabilitation. We hypothesized that older age may influence the incidence of particular complications, such as UGIB or infections, in these patients. Thus, the objective of this study was to retrospectively investigate the effect of age on the occurrence of various complications among first-time patients with stroke from the acute stage to subsequent hospitalization in the rehabilitation ward.

## Methods

### Participants and assessment procedures

The medical records of patients consecutively admitted to the rehabilitation department between July 2002 and June 2012 were retrospectively reviewed. Study patients met the following inclusion criteria: (a) confirmed, acute, first-time stroke on the basis of the World Health Organization criteria at our hospital; (b) limb motor deficits; and (c) transfer to rehabilitation ward from an acute ward during first-time stroke hospitalization. The Institutional Review Board for Human Studies at Chang Gung Memorial Hospital approved the study protocol.

Demographic data, including age, gender, mean length of stay in the rehabilitation ward, and duration of stay in the acute ward during first-time stroke hospitalization, were recorded. In addition, data on stroke type (ischemic or hemorrhagic), location (left, right, or bilateral), and improvement in Brunnstrom’s stage of motor recovery (BMR) in the affected limbs following transfer to the rehabilitation ward were also collected. Brain computed tomography and/or magnetic resonance imaging performed at stroke onset were reviewed for stroke type and location. BMR staging classifies the sequential motor recovery of stroke patients into six stages, with the recovery being better as the stage increases. Improvement in BMR is defined as any change from the lower to higher stage of the affected limbs (proximal upper limb, distal upper limb, or lower limb) between transfer to the rehabilitation ward and discharge.

Post-stroke neurological deficits such as neurogenic bladder, speech problems (aphasia or dysarthria), dysphagia, and numbness were recorded according to medical records. A complication was defined as one medical event or neurological problem requiring a doctor’s evaluation or management. Data regarding complications, including UGIB, constipation, fever, infection, fall, seizure, depression, anxiety, stroke progression, pressure ulcer, musculoskeletal pain, uncontrolled hypertension, uncontrolled diabetes mellitus (DM), dermatitis, scabies, deep vein thrombosis, pulmonary embolism, electrolyte imbalance (hyponatremia, hypernatremia, hypokalemia, and hyperkalemia), hypoalbuminemia, allergic reaction, and acute coronary syndrome, were obtained from patient medical records. Infections recorded included pneumonia, symptomatic urinary tract infection (UTI), upper respiratory tract infection, cellulitis, bloodstream infection (not concurrent with any other infections), herpes zoster, surgical wound infection (after craniotomy or craniectomy), and central nervous system infection. Depression and anxiety were diagnosed by a psychiatrist when the symptoms met the Diagnostic and Statistical Manual of Mental Disorders criteria, fourth edition. Musculoskeletal pain included that of the shoulder, knee, hip, wrist, ankle, back, and neck. Uncontrolled hypertension was defined as elevated blood pressure requiring a cardiologist consultation for adjustment of antihypertensive therapy. Uncontrolled DM was defined as elevated blood glucose requiring an endocrinologist consultation for the adjustment of oral hypoglycemic/insulin therapy. Hyponatremia and hypernatremia were defined as serum sodium <134 and >148 mEq/L, respectively; hypokalemia and hyperkalemia as serum potassium <3.6 and >5.0 mEq/L, respectively; and hypoalbuminemia as serum albumin <3.5 g/dL.

In general, individuals are defined as elderly when they are aged >65 years (retirement age in Taiwan) and younger old when they are aged 65–75 years [20]. In this study, cutoff points for comparison were set at 65 and 75 years, and patients were classified into three age groups: <65 years, young; 65 to <75 years, younger old; and ≥75 years, older old.

Subsequent hospitalization was defined as readmission to the rehabilitation ward. The incidence of each complication in the rehabilitation ward for each patient during subsequent hospitalizations was defined as the percentage of the complication occurring relative to the number of readmissions (e.g., pneumonia occurring twice in 10 readmissions = incidence of pneumonia of 20% for each patient). The incidence of each complication during subsequent hospitalizations in each age group was defined as the average of the incidence of each complication of all patients in each age group. In the case of a patient having recurrent stroke, complications and readmissions to the rehabilitation ward were not included in the calculations.

### Statistical analysis

SPSS 12.0 for Windows was used for analysis. Length of stay (days) in the acute and rehabilitation wards was compared using paired *t*-tests, and differences in incidence of various complications in these wards was analyzed using McNemar’s test.

One-way analysis of variance was used to compare continuous variables among the three groups, and Bonferroni correlation for post-hoc comparison between any two groups. Chi-square and Fisher’s exact tests were employed to compare categorical variables. A p-value of <0.05 was considered statistically significant.

## Results

**Figure 1:**
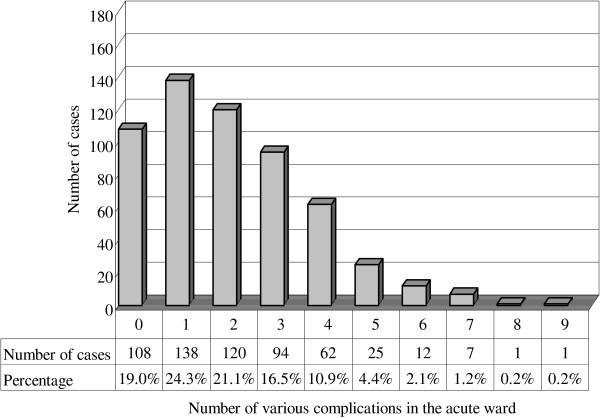
**The number of cases and various complications in the acute ward.** The numbers on the X-axis correspond to the following: 0 = the sum of the complication types is zero, 1 = the sum of the complication types is one, and so on. The major numbers of various complications in the acute ward were 0, 1, 2, and 3, resulting from 19.0%, 24.3%, 21.1%, and 16.5% of total patients, respectively.

**Figure 2:**
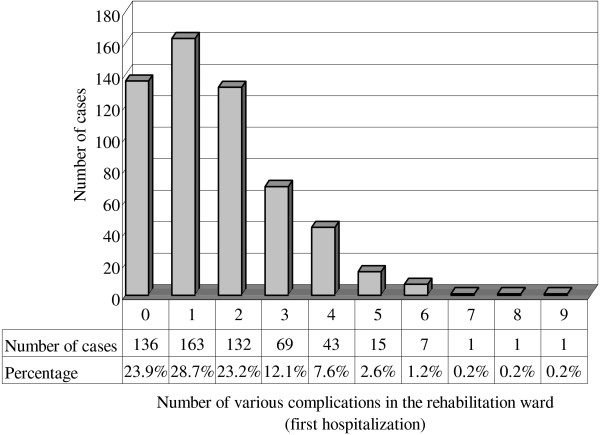
**The number of cases and various complications in the rehabilitation ward.** The numbers on the X-axis correspond to the following: 0 = the sum of the complication types is zero, 1 = the sum of the complication types is one, and so on. The major numbers of various complications in the rehabilitation ward were 0, 1, 2, and 3, resulting from 23.9%, 28.7%, 23.2%, and 12.1% of total patients, respectively.

Between July 2002 and June 2012, among 719 stroke patients hospitalized in the rehabilitation ward, we enrolled 568 who met the study criteria (283 males, 285 females; mean age at first-time stroke onset 65.71 ± 13.33 years; Table 1). In the acute ward, patients experienced less shoulder pain, neck pain, knee pain, other musculoskeletal pain, dermatitis, and hyperkalemia than in the rehabilitation ward during initial stay. However, in the acute ward, patients experienced more neurogenic bladder, speech problems, dysphagia, at least one complication, fever, pneumonia, uncontrolled hypertension, hypokalemia, and hypoalbuminemia than in the rehabilitation ward during initial stay (see Table 1). Associations between the number of cases and number of various complication types (among 30 different complication types evaluated) in the acute and rehabilitation wards (first hospitalization) are presented in Figures 1 and 2, respectively. The correlations between age and number of complication types in both the acute (p = 0.260; r = −0.047) and rehabilitation wards (first hospitalization; p = 0.126; r = 0.064) showed no significance.

**Table 1:** Clinical characteristics and comparison of neurological deficits and complications during initial hospitalization in acute and rehabilitation wards

	**Acute ward**	**Rehabilitation ward**	**p value**
Total	568	568	N/A
Gender			
Males	283	283	N/A
Females	285	285	N/A
Age (years)	65.71 ± 13.33	65.71 ± 13.33	N/A
Length of stay (days)	22.15 ± 11.57	25.29 ± 11.72	<0.001^a^
Neurological deficits	Case numbers		
Neurogenic bladder	275 (48.4)	254 (44.7)	0.01^a^
Speech problems	410 (72.2)	377 (66.4)	<0.001^a^
Dysphagia	397 (69.9)	372 (65.5)	0.012^a^
Numbness	74 (13.0)	55 (9.7)	0.078
Complications	Case numbers		
At least one complication	460 (81.0)	432 (76.1)	0.04^a^
UGIB	59 (10.4)	76 (13.4)	0.1
Constipation	157 (27.6)	161 (28.3)	0.845
Fever	203 (35.7)	91 (16)	<0.001^a^
Pneumonia	106 (18.7)	29 (5.1)	<0.001^a^
Symptomatic UTI	111 (19.5)	97 (17.1)	0.327
Other infections	25 (4.4)	33 (5.8)	0.332
Fall	6 (1.1)	13 (2.3)	0.167
Seizure	18 (3.2)	9 (1.6)	0.108
Depression	50 (8.8)	38 (6.7)	0.207
Anxiety	5 (0.9)	7 (1.2%)	0.774
Stroke progression	6 (1.1)	7 (1.2)	1.000
Pressure ulcers	4 (0.7)	5 (0.9)	1.000
Shoulder pain	15 (2.6)	132 (23.2)	<0.001^a^
Neck pain	13 (2.3)	27 (4.8)	0.038^a^
Back pain	15 (2.6)	17 (3.0)	0.856
Knee pain	13 (2.3)	40 (7.0)	<0.001^a^
Other musculoskeletal pain	24 (4.2)	52 (9.2)	0.001^a^
Uncontrolled hypertension	23 (4.0)	11(1.9)	0.029^a^
Uncontrolled DM	6 (1.1)	5(0.9)	1.000
Dermatitis	21(3.7)	41 (7.2)	0.005^a^
Scabies	1 (0.2)	1 (0.2)	1.000
Deep vein thrombosis	0 (0)	1 (0.2)	N/A^b^
Pulmonary embolism	1 (0.2)	4 (0.7)	0.993
Hyponatremia	37 (6.2)	32 (5.6)	0.597
Hypernatremia	0 (0)	1 (0.2)	N/A^b^
Hypokalemia	178 (31.3)	16 (2.8)	<0.001^a^
Hyperkalemia	8 (1.4)	22 (3.9)	0.013^a^
Hypoalbuminemia	60 (10.6)	17 (3.0)	<0.001^a^
Allergy	5 (0.9)	4 (0.7)	1.000
Acute coronary syndrome	1 (0.2)	3 (0.5)	0.625

Data are expressed as mean ± standard deviation for age and length of stay, and as case numbers for gender, neurological deficits and complications. Figures in parentheses are percentage.

^a^p < 0.05. ^b^The p value was not obtained using McNemar’s test. UGIB = upper gastrointestinal bleeding;

UTI = urinary tract infection; DM = diabetes mellitus.

The results of comparing clinical characteristics, neurological deficits, and complications among the three groups after transfer from the acute ward to the rehabilitation ward are shown in Table 2. The incidence of neurogenic bladder, dysphagia, UGIB, and symptomatic UTI was significantly lower in the young group than in the younger old and older old groups (see Table 2).

**Table 2:** Clinical characteristics, neurological deficits, and complications among three age groups after transfer of patients from acute to rehabilitation ward

	**Age group**			**p value**
	**<65 years (n = 218)**	**65–75 years (n = 195)**	**≥75 years (n = 155)**	
Male	148 (67.9)	83 (42.6)	52 (33.5)	<0.001^a^
Ischemic stroke	88 (40.4)	113 (57.9)	112 (72.3)	<0.001^a^
Hemorrhagic stroke	130 (59.6)	82 (42.1)	43 (27.7)	<0.001^a^
Brain lesion				
Left brain	113 (51.8)	88 (45.1)	67 (43.2)	0.202
Right brain	97 (44.5)	104 (53.3)	81 (52.3)	0.150
Bilateral brain	8 (3.7)	3 (1.5)	7 (4.5)	0.249
Stroke-induced impaired consciousness	136 (62.4)	105 (53.8)	73 (47.1)	0.012^a^
Mean acute ward stay, days	20.8 ± 10.11	23.01 ± 12.59	22.96 ± 12.03	0.09
Mean rehabilitation ward stay, days	25.42 ± 10.65	24.99 ± 12.23	25.48 ± 12.56	0.907
Improvement in BMR stage staying in rehabilitation ward	59 (27.1)	38(19.5)	35(22.6)	0.186
Neurological deficits				
Neurogenic bladder	80 (36.7)	94 (48.2)	80 (51.6)	0.008^a^
Speech problems	145 (66.5)	130 (66.7)	102 (65.8)	0.984
Dysphagia	129 (59.2)	133 (68.2)	110 (71.0)	0.038^a^
Numbness	25 (11.5)	17 (8.7)	13 (8.4)	0.522
Complications				
At least one complication	160 (73.4)	152 (77.9)	120 (77.4)	0.499
UGIB	9 (4.1)	35 (17.9)	32 (20.6)	<0.001^a^
Constipation	59 (27.1)	61 (31.3)	41 (26.5)	0.528
Fever	32 (14.7)	31 (15.9)	28 (18.1)	0.679
Pneumonia	7 (3.2)	9 (4.6)	13 (8.4)	0.076
Symptomatic UTI	25 (11.5)	41 (21.0)	31 (20.0)	0.019^a^
Other infections	15 (6.9)	12 (6.2)	6 (3.9)	0.458
Fall	8 (3.7)	2 (1.0)	3 (1.9)	0.189
Seizure	4 (1.8)	4 (2.1)	1 (0.6)	0.539
Depression	12 (5.5)	18 (9.2)	8 (5.2)	0.214
Anxiety	4 (1.8)	3 (1.5)	0 (0)	0.255
Stroke progression	3 (1.4)	3 (1.5)	1 (0.6)	0.731
Pressure ulcers	0 (0)	3 (1.5)	2 (1.3)	0.202
Shoulder pain	51 (23.4)	43 (22.1)	38 (24.5)	0.861
Neck pain	11 (5.0)	8 (4.1)	8 (5.2)	0.869
Back pain	8 (3.7)	4 (2.1)	5 (3.2)	0.616
Knee pain	11 (5.0)	12 (6.2)	17 (11.0)	0.074
Other musculoskeletal pain	24 (11.0)	17 (8.7)	11 (7.1)	0.420
Uncontrolled hypertension	6 (2.8)	2 (1.0)	3 (1.9)	0.200
Uncontrolled DM	2 (0.9)	2 (1.0)	1 (0.6)	0.928
Dermatitis	19 (8.7)	12 (6.2)	10 (6.5)	0.550
Scabies	0 (0)	0 (0)	1 (0.6)	0.263
Deep vein thrombosis	0 (0)	0 (0)	1 (0.6)	0.263
Pulmonary embolism	1 (0.5)	1 (0.5)	2 (1.3)	0.591
Hyponatremia	8 (3.7)	11 (5.5)	13 (8.4)	0.150
Hypernatremia	0 (0)	0 (0)	1 (0.6)	0.263
Hypokalemia	6 (2.8)	7 (3.6)	3 (1.9)	0.648
Hyperkalemia	5 (2.3)	7 (3.6)	10 (6.5)	0.118
Hypoalbuminemia	5 (2.3)	5 (2.6)	7 (4.5)	0.421
Allergy	2 (0.9)	1 (0.5)	1 (0.6)	0.882
Acute coronary syndrome	2 (0.9)	0 (0)	1 (0.6)	0.426

Data are expressed as case numbers. Figures in parentheses are percentage.

^a^p < 0.05. BMR = Brunnstrom’s stages of motor recovery. UGIB = upper gastrointestinal bleeding.

UTI = urinary tract infection; DM = diabetes mellitus.

A total of 150 patients underwent subsequent hospitalization in the rehabilitation ward (Table 3). The incidence of UGIB was significantly lower in the young group than in the younger old and older old groups (differences were between the young and younger old groups and the young and older old groups). The incidence of symptomatic UTI was significantly higher in the older old group than in the younger old and young groups (differences were between the young and older old groups and the younger old and older old groups; see Table 3).

**Table 3:** Incidence (%) of complications in patients during readmission to rehabilitation ward among the three age groups

**Complications**	**Age group**			**p value**
	**<65 years (n = 88)**	**65–75 years (n = 38)**	**≥75 years (n = 24)**	
UGIB	0.19 ± 1.78	8.04 ± 24.70	8.33 ± 24.08	0.011^a^
Constipation	16.17 ± 32.59	25.58 ± 39.82	18.75 ± 35.55	0.385
Fever	7.81 ± 22.55	8.04 ± 21.09	12.50 ± 30.40	0.680
Pneumonia	1.31 ± 10.77	2.63 ± 16.22	8.33 ± 24.08	0.131
Symptomatic UTI	3.14 ± 15.60	9.21 ± 25.91	29.17 ± 44.03	<0.001^a^
Other infections	0.91 ± 4.32	1.32 ± 8.11	2.08 ± 10.21	0.737
Fall	0.38 ± 2.13	1.61 ± 8.26	0	0.277
Seizure	1.36 ± 0.96	0	0	0.542
Depression	2.70 ± 15.04	6.14 ± 23.06	8.33 ± 28.23	0.391
Anxiety	1.14 ± 10.66	0	0	0.706
Stroke progression	0	0	0	N/A^b^
Pressure ulcers	0.10 ± 0.97	1.75 ± 8.48	6.25 ± 22.42	0.028^a^
Shoulder pain	22.02 ± 33.56	26.90 ± 40.90	27.08 ± 44.18	0.727
Neck pain	3.52 ± 13.13	3.94 ± 17.94	4.17 ± 20.41	0.980
Back pain	6.60 ± 20.80	4.82 ± 18.55	11.46 ± 29.47	0.498
Knee pain	7.34 ± 22.33	4.24 ± 17.96	5.21 ± 20.82	0.727
Other musculoskeletal pain	13.13 ± 25.06	3.80 ± 16.99	13.54 ± 30.38	0.122
Uncontrolled hypertension	1.14 ± 10.66	2.63 ± 16.22	0	0.663
Uncontrolled DM	0.38 ± 3.55	0	0	0.706
Dermatitis	8.83 ± 19.78	5.99 ± 19.73	0	0.107
Scabies	0.23 ± 1.81	4.97 ± 18.53	0	0.027^a^
Deep vein thrombosis	0	0	4.17 ± 20.41	0.072
Pulmonary embolism	0	0	0	N/A^b^
Hyponatremia	0.19 ± 1.78	7.16 ± 23.74	6.25 ± 22.42	0.029^a^
Hypernatremia	0	4.39 ± 2.70	0	0.230
Hypokalemia	3.94 ± 18.35	0	6.25 ± 22.42	0.309
Hyperkalemia	0.10 ± 0.97	2.63 ± 11.31	2.08 ± 10.21	0.135
Hypoalbuminemia	1.14 ± 10.66	3.95 ± 17.94	2.08 ± 10.21	0.530
Allergy	1.86 ± 1.39	0	0	0.579
Acute coronary syndrome	0	0	0	N/A^b^

Data are expressed as mean values ± standard deviation.

^a^p < 0.05. ^b^The p value was not obtained using One-way ANOVA. UGIB = upper gastrointestinal bleeding;

UTI = urinary tract infection; DM = diabetes mellitus.

## Discussion

Age can affect the occurrence of particular stroke complications. UGIB and symptomatic UTI were found to be the complications affected by age in both first and subsequent admissions to the rehabilitation ward. The reported incidence of UGIB during initial stroke rehabilitation ranges from 3.4%, as reported by Kitisomprayoonkul et al. [8], to 8.6%, as reported by Doshi et al. [10]; however, our results for the initial admission in the rehabilitation ward (13.4%) were relatively high compared with the published results. We believe that the incidence of UGIB was higher in our study compared with those reported in the previous two studies mainly due to differences in the study methods. Kitisomprayoonkul’s study [8] did not enroll stroke patients in the acute phase because their mean onset-to-admission period was 65.95 days. Doshi’s study [10] enrolled only 140 patients and the diagnosis of UGIB was according to the coffee ground aspirate from the nasogastric tube, and did not include esophagogastroduodenoscopy findings. However, documentation for the diagnosis of UGIB in our hospital is based on either the coffee ground aspirate from the nasogastric tube or ulcers, erosions, or bleeding sources proven by esophagogastroduodenoscopy. One previous article [17] found that 20.5% of stroke patients aged ≥65 years experienced UGIB during their first stay in the rehabilitation ward. In that article, the mean age of UGIB patients was higher (75.4 years) than those of without UGIB (72.9 years). However, there was no difference in UGIB incidence between the younger old and older old groups in our study, possibly because of the choice of cutoff points. In a population-based study [21], Longstreth illustrated that the annual incidence in the general population for acute UGIB was only 0.102%. Compared to the incidence rate of UGIB during initial admission in the rehabilitation ward in our study, acute stroke patients appeared to have higher risk for UGIB than the general population. However, the question still remains; how does age effect the occurrence of UGIB in acute stroke patients? Schaller et al. [22] reported that cerebral ischemia may lead to an interruption of the axis between the central nervous and gastrointestinal systems, which could cause gastrointestinal hemorrhage or dysmotility. In addition, seroprevalence of *Helicobactor pylori* increased from 21% in the third decade of life to 50% in the eighth decade of life [23], and *Helicobactor pylori* infection is a risk factor for gastric and duodenal ulcers. However, whether *Helicobactor pylori* infection is the only cause of pathophysiology, or if it works in combination with other factors to influence the effects of age on the occurrence of UGIB in stroke patients still needs to be studied.

Younger stroke patients (<65 years) were less likely to have symptomatic UTI during their first stay in rehabilitation, and in older patients (>75 years) this was more likely to occur during subsequent stays. Stott et al. [24] showed that UTI is associated with increasing age by decade after stroke, whereas Luk et al. [25] enrolled acute stroke patients undergoing rehabilitation and stratified them into age groups of <65, ≥65 to <80, and ≥80 years, with no difference observed in UTI incidence among the three age groups. Another study [19] found no differential effect of age between elderly (≥65 years) first-time stroke patients with and without UTI during their stay in the rehabilitation ward. In contrast, our study focused not only on the status of first-time stroke patients in the rehabilitation ward but also on collecting data from subsequent readmissions. We consider that different enrolment criteria and stratification by age group in our study may have led to the difference in the results from other studies on the effect of age in these patients. One study illustrated that an excessive anti-inflammatory response is a key facilitating factor in the development of infection, and this immunological response could result from an adaptive mechanism to brain ischemia [26]. We postulate that some acute stroke patients that have urinary retention will require urinary catheterization, and urinary catheterization may predispose these patients to symptomatic UTI [24]. Moreover, asymptomatic bacteriuria is prevalent among the elderly [27] and could be another facilitating factor for symptomatic UTI in older age patients. However, whether these factors or other factors influence the impact of age on the occurrence of symptomatic UTI in these patients needs further research.

Sum of the complication types >3 included <20% of the patients in both the acute and first hospitalization in rehabilitation ward groups. It appears that the trend of the association between case numbers and number of complication types was similar in both the acute and rehabilitation ward (first hospitalization) groups. For stroke patients in the rehabilitation ward, Hung’s retrospective study [6] showed that the presence of at least one complication was not significantly associated with older age. Our study showed similar results when age group and with/without at least one complication during the initial stay in the rehabilitation ward were compared. We consider that the reason for this result was that most complications were not affected by age during this period, i.e., no significant differences.

Knoflach et al. [28] found age to be a significant inverse predictor of good outcome (modified Rankin Scale score) after ischemic stroke, but no hemorrhagic stroke patients were enrolled. Luk et al. [25] demonstrated that age is not an independent predictor of good outcome (by alteration in functional independent measure) for stroke rehabilitation. In our study, no difference was noted among the three age groups with respect to BMR stage improvement following transfer to the rehabilitation ward. We believe that different outcome measurements may yield different results; therefore, other tools could be used in further studies to illustrate various outcomes in these patients.

Neurogenic bladder and dysphagia were significantly improved from the acute to the rehabilitation wards, with the incidence of both conditions being lower in patients aged <65 than in those aged >65 years. Although the incidence of speech problems also decreased from the acute to the rehabilitation wards, lower age had no effect. Numbness followed a different pattern, with slow recovery and no effect of age on incidence with respect to transfer from acute to rehabilitation ward.

The incidence of shoulder pain (23.2%) ranked as the second highest complication in stroke patients first transferred to the rehabilitation ward, this being similar to the results of Kuptniratsaikul (19%) [3] and McLean (24%) [4]; in addition, for first-time stroke patients, this incidence in the acute ward (2.6%) was much lower than that in the rehabilitation ward. However, the reported incidence of shoulder pain in post-stroke hemiplegia ranges from 34% [29] to 84% [30], with many pathologies (e.g., capsulitis, shoulder subluxation, impingement syndrome, rotator cuff injury, and shoulder–hand syndrome). The incidence of shoulder pain in the acute ward in our study may have been underestimated because of the retrospective method employed. The pain may not have been induced because patients will probably not move the joint, following acute stroke in the acute ward. This will possibly decrease the actual case numbers of shoulder pain documented in the medical charts.

In the elderly, increased skin fragility with age may be a risk for skin injury, including pressure ulcers. Although the incidence of pressure ulcers showed no difference among the three age groups during initial stay in the rehabilitation ward in our study (total, *n* = 5), this was significantly higher in patients >75 years of age during subsequent readmission to the rehabilitation ward (*n* = 5). According to Wang’s report [31], the incidence of pressure ulcer in an inpatient rehabilitation facility was 5.23%, and these patients had lower motor gain and longer length of stay than those without pressure ulcers. Case numbers with pressure ulcer in our study were too low for further analysis, and enrolment of more patients would be required for studying the association between low incidence of complication and outcome.

Hyponatremia is a common electrolyte imbalance in hospitalized patients and is used as an indicator for outcome in acute coronary syndrome [32] and stroke [33]. However, hyponatremia has not been discussed with respect to patients undergoing stroke rehabilitation. In the present study, the incidence of hyponatremia was lower in patients aged >65 years than in those aged >65 years readmitted to the rehabilitation ward. Because checking of electrolytes was not a routine procedure for such patients, we consider that the number of cases of hyponatremia may have been underestimated. A prospective study involving strict electrolyte level monitoring may improve the reliability of the data.

Our study mainly focused on the effect of age on various complications, but other factors may also contribute to their incidence. The major limitation of this study is the limited case numbers, with the low incidence of some complications limiting the analytical proof with respect to age distribution. Another limitation is the retrospective study design which may not reflect the true incidence of complications due to errors in documentation of classification. In addition, BMR staging used as the only outcome measurement may have been insufficient for the outcome analysis. Despite these limitations, this is the first study to elucidate the overall complication rates according to age for inpatient stroke rehabilitation. Future studies should enroll higher patient numbers for further analysis and utilize more outcome measurements in a prospective manner.

## Conclusions

To summarize, age can affect the incidence of UGIB and symptomatic UTI in stroke patients in the rehabilitation ward. Patients under 65 years were less likely to have UGIB, whereas those over 75 years were more likely to have symptomatic UTI than those under 65 years. No difference with respect to improvement in limb movement was found among age groups with respect to initial transfer to the rehabilitation ward. These findings will help clinicians to understand the effects of age on various complications during different stages of inpatient rehabilitation in stroke patients.

## Abbreviations

UGIB: Upper gastrointestinal bleeding; UTI: Urinary tract infection; BMR: Brunnstrom’s stage of motor recovery; DM: Diabetes mellitus.

## Competing interests

The authors’ declare that they have no competing interests.

## Authors’ contributions

Chien-Min Chen, Hung-Chih Hsu, and Chia-Hao Chang conceived project. Chu-Hsu Lin, Kai-Hua Chen, Wei-Chi Hsieh, and Wen-Ming Chang were responsible for data collection and management. Chia-Hao Chang was responsible for data analyses. Chien-Min Chen wrote the first draft of the article and all authors contributed to the development of the manuscript. All authors read and approved the final manuscript.

## Pre-publication history

The pre-publication history for this paper can be accessed here:

http://www.biomedcentral.com/1471-2318/14/41/prepub
